# Sedation and analgesia doses do not differ across demographic factors in pediatric cardiac surgery patients

**DOI:** 10.3389/fped.2025.1577461

**Published:** 2025-07-16

**Authors:** Nikki R. Lawson, Barbara Jo Achuff, Ashish Ankola, Danielle Guffey, Keila Lopez, Natasha Afonso

**Affiliations:** ^1^ Department of Pediatrics, Division of Critical Care Medicine, Baylor College of Medicine, Houston, TX, United States; ^2^ Department of Pediatrics, Division of Critical Care Medicine, Vanderbilt University Medical Center, Nashville, TN, United States; ^3^ Institute for Clinical & Translational Research, Baylor College of Medicine, Houston, TX, United States; ^4^ Department of Pediatrics, Division of Cardiology, Baylor College of Medicine, Houston, TX, United States

**Keywords:** disparities health, sedation, analgesia & critical care, critical care, cardiac surgery

## Abstract

**Importance:**

Disparities in congenital heart disease, pediatric intensive care unit (ICU) outcomes, and acute pain control are common. The impact of patient race, ethnicity, and language on postoperative sedation and analgesia for pediatric patients undergoing cardiac surgery is unknown.

**Objective:**

This study aims to investigate whether there are differences in sedation and analgesia in patients undergoing cardiac surgery according to patient race, ethnicity, and language in a study site that uses protocolized postoperative sedation and analgesia. We hypothesized that non-white and patients who prefer a language other than English would have differences in total doses of sedation compared with their white and English-speaking counterparts.

**Design:**

This is a single-center, retrospective, observational cohort of pediatric patients admitted postoperatively to the cardiac ICU.

**Setting:**

The study took place in an urban, quaternary, academic pediatric referral center.

**Participants:**

All pediatric patients, age 0–18 years, admitted to the cardiac ICU following their index cardiac surgery from 7/1/2018 to 6/30/2022 were included. Patients requiring renal replacement therapy, non-cardiac surgery during the same admission, tracheostomy, preoperative mechanical ventilation, or mechanical circulatory support prior to or following cardiac surgery were excluded.

**Exposures:**

Exposure variables included patient race and ethnicity, preferred language, age, gender, Society of Thoracic Surgeons-European Association for Cardiothoracic Surgery category, use of cardiopulmonary bypass, duration of postoperative mechanical ventilation, and delayed sternal closure (DSC).

**Main outcome and measure:**

The primary outcome was weight-adjusted doses of opioids in morphine equivalents, benzodiazepines in midazolam equivalents, and dexmedetomidine received in the first 72 h postoperatively.

**Results:**

There were 1,794 postoperative admissions. 100% of patients received opioids, 42.5% received benzodiazepines, and 85% received dexmedetomidine. There were no differences in opioid, benzodiazepine, or dexmedetomidine doses according to patient race and ethnicity or preferred language. Patient race, ethnicity, and language were not associated with opioid or benzodiazepine dose in multivariable quantile regression. Multivariable regression for dexmedetomidine demonstrated similar results with age also being inversely correlated.

**Conclusions:**

and Relevance: Doses of postoperative sedation and analgesia are not correlated with patient race, ethnicity, and language. Factors that were associated with differences in medication doses are reflective of postoperative acuity and are expected. Protocolized sedation and analgesia may be responsible for the lack of differences seen in this study when compared with other studies in similar settings. Future studies should compare protocolized and non-protocolized sedation and analgesia to further evaluate the protective effects of protocols against bias in clinical settings.

## Introduction

Social determinants of health are known to effect health outcomes for pediatric patients and are often the root cause of health inequities among racially, ethnically, and linguistically diverse groups. These effects have been specifically identified in outcomes for patients in pediatric intensive care units (ICU), with congenital heart disease (CHD), and with acute pain complaints (1–5). For pediatric patients admitted to the ICU, differences include higher rates of admission and longer lengths of stay for patients with higher rates of poverty (1). Higher rates of mortality have been reported for Hispanic patients, patients from lower socioeconomic backgrounds, and patients without insurance (6–8). Black patients are also overrepresented in those admitted to the PICU relative to the general population, even when controlling for insurance status and initial illness severity (1). Additionally, patients who prefer a language other than English encounter elevated hospitalization costs, increased incidence of serious adverse events, and more frequent transfers to the ICU, all while expressing diminished satisfaction with communication from medical teams during their hospitalization (2, 4, 9–11).

Similar disparities are seen in pediatric patients with CHD. Though there have been marked advancements in surgical outcomes, non-Hispanic white children have experienced greater reductions in mortality rate than their Black counterparts (3). Similarly, Black children also exhibit higher rates of postoperative complications after cardiac surgery (12, 13).

Regarding acute pain management, non-white children with long bone fractures or abdominal pain in emergency departments are less likely to achieve optimal pain reduction during their visits (14, 15). Results from the perioperative period are mixed with some studies finding differences in post- or intraoperative opioid administration or receipt of regional anesthesia, but equivocal results are reported (16–18).

Several studies have evaluated the use of sedation protocols that are driven by nursing assessments of level of sedation and pain with medication doses adjusted accordingly (19–22). Overall, these studies suggest that sedation targets using validated scores can safely reduce doses of benzodiazepines while not effecting duration of mechanical ventilation, PICU LOS, pain scores, or rates of unplanned extubations. While these studies have demonstrated the safety of protocolized sedation, they have not evaluated these protocols through the lens of health equity. A secondary analysis of the RESTORE trial, one of the largest randomized studies that has evaluated protocolized sedation in pediatrics, found no differences in doses of sedation and analgesia according to race and ethnicity in both the control and intervention arms (Nathaly 2021). Importantly, this study did not evaluate the contribution of language and was limited to the original study cohort of pediatric patients intubated for respiratory failure.

Despite knowledge of disparities in pediatric ICUs, CHD, and acute pain management, there is a paucity of knowledge on the impact of race, ethnicity, and language on sedation and analgesia for postoperative cardiac patients in the immediate postoperative period.

Given what is known about disparities in PICU outcomes and sedation and analgesia, we hypothesize that following cardiac surgery, differences will exist in the cumulative doses of medications received for sedation and analgesia among non-white and non-English speaking patients compared to their white and English-speaking counterparts. The primary aim of this study is to compare the weight-adjusted dose of medications received for sedation and analgesia following cardiac surgery based on patient race, ethnicity, and language in a study site that uses protocolized post-operative sedation and analgesia. The secondary aim is to evaluate patient outcomes such as ventilator days, rate of unplanned extubations, cardiac arrest, and mortality.

## Methods

This study is a single-center, retrospective observational cohort of pediatric patients (age 0–18 years) who were admitted to the cardiac ICU following cardiac surgery from July 1, 2018, to June 30, 2022. Patients were excluded if they required renal replacement therapy, had non-cardiac surgery during the same admission, pre-existing tracheostomy, preoperative mechanical ventilation, or required mechanical circulatory support prior to or following the cardiac surgery. Patients who had pain managed with exclusively patient-controlled analgesia (PCA) were also excluded due to incomplete data collection. The study site is a large, urban, academic pediatric hospital system in the United States. A standardized sedation and pain management protocol was implemented in the cardiac ICU in 2017 and was adopted prior to the study. The pathways give providers starting medications and doses and include the use of documented State Behavior Scale (SBS) for sedation as well as validated scores for pain based on age to allow nurses and practitioners to titrate sedative and pain medications to desired effect.

Data for doses of sedation and analgesia, race, ethnicity, and language were obtained from the electronic medical record and all other information from the local Pediatric Cardiac Critical Care Consortium (PC4) registry database. Both databases were used equally for all patients. The study was approved by the Baylor College of Medicine Institutional Review Board and informed consent for this historical study was waived.

Exposure variables included combined patient race and ethnicity, patient preferred language, age, gender, Society of Thoracic Surgeons-European Association for Cardio-Thoracic Surgery (STAT) category, use of cardiopulmonary bypass (CPB), duration of postoperative mechanical ventilation, and delayed sternal closure (DSC). Race, ethnicity, and preferred language are either self-reported by parents or documented by staff upon admission to the hospital or an inpatient setting. Patients with missing race and ethnicity, or language data were excluded from analysis (*n* = 10).

The primary outcome was weight-adjusted doses of opioids, benzodiazepines, and dexmedetomidine received in the first 72 h postoperatively. By using the first 72 postoperative hours, the focus remains on treatment of acute, perioperative pain and avoiding variations that occur due to length of stay, comorbidities, or other complications. Opioids were converted to morphine milligram equivalents and benzodiazepines were converted to midazolam milligram equivalents for the purpose of comparison. The secondary outcomes were duration of mechanical ventilation, unplanned extubations, cardiac arrest, and mortality as collected in PC4.

Patient characteristics and non-parametric outcomes are described using mean with standard deviation (SD), median with interquartile range (IQR), and frequency with percentage. Wilcoxon rank sum test, Chi square test, and Fisher's exact test were used to compare categorical characteristics. Unadjusted and multivariable quantile regression were performed to assess the association between characteristics and medication doses. A *p*-value of <0.05 was considered statistically significant for all comparisons and all analyses were performed using Stata v18 (College Station, TX).

## Results

**Figure 1:**
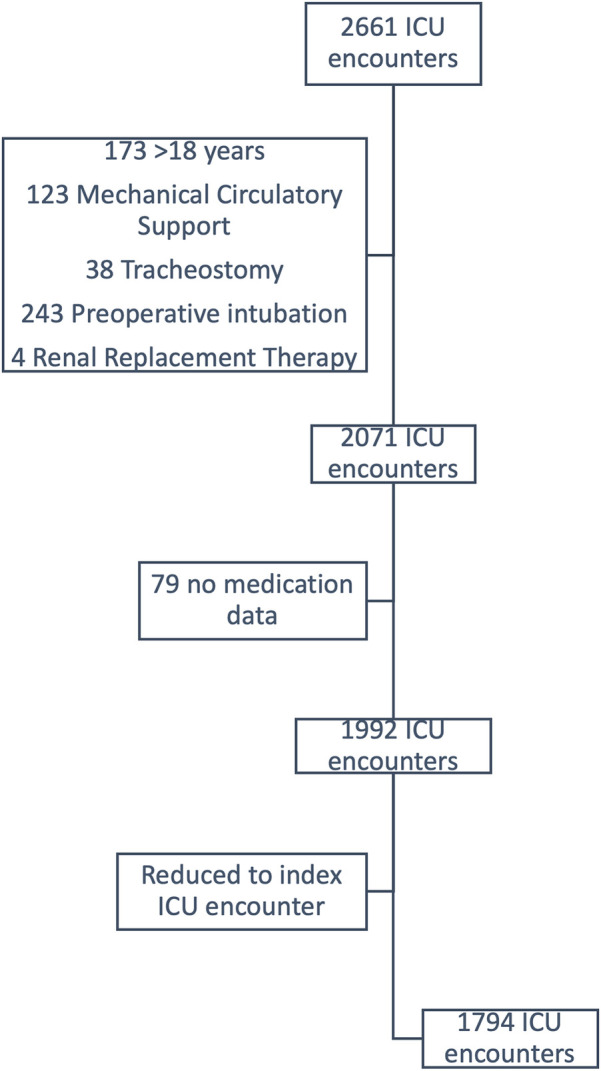
Explanation of encounter exclusions.

The cohort included 2,661 postoperative CICU encounters. After applying exclusion criteria, there were 1,794 remaining encounters (Figure 1). Demographics of the cohort are found in Table 1. Notably, the median age of the cohort upon admission was 2 years (IQR 0–6 years) and the majority were male (*n* = 963, 53.7%). Despite 702 (39.4%) of patients identifying as Hispanic white, only 235 (13.1%) preferred Spanish as a primary language. Most surgeries were classified as STAT 1 (*n* = 995, 55.8%) and only 57 (3.2%) as STAT 5. Comparisons in DSC, STAT category distribution, operation requiring CPB, or rates of postoperative mechanical ventilation according to patient race and ethnicity or language revealed no differences (Table 2).

**Table 1:** Patient demographics.

	Median	IQR
	*n* = 1,794	
Hospital admit age (years)	2	0, 6
	*N*	%
Race and Ethnicity		
Non-Hispanic White	706	39.6%
Non-Hispanic Black	232	13.0%
Hispanic White	702	39.4%
Hispanic Black	17	1.0%
Other	126	7.1%
Gender		
Female	831	46.3%
Male	963	53.7%
Language		
English	1,520	84.7%
Spanish	235	13.1%
Other	39	2.2%
Insurance		
Public	945	52.7%
Private	805	44.9%
None/Self pay	43	2.4%
STAT Category		
1	995	55.8%
2	318	17.8%
3	254	14.2%
4	160	9.0%
5	57	3.2%
Delayed sternal closure	92	5.1%
Post-operative Mechanical Ventilation	1,076	60.0%

**Table 2:** Comparison of STAT category, DSC, CPB, and post-operative mechanical ventilation according to patient race, ethnicity, and language.

	STAT category 4 or 5			Delayed sternal closure			Cardiopulmonary bypass			Post-operative mechanical ventilation		
	*N*	%	*p* value	*N*	%	*p* value	*N*	%	*p* value	*N*	%	*p* value
Race and Ethnicity												
Non-Hispanic White	102	14.50%	0.08	39	5.5%	0.66	616	87.3%	0.67	426	60.3%	0.55
Non-Hispanic Black	19	8.20%		9	3.9%		204	87.9%		138	59.5%	
Hispanic White	80	11.50%		35	5.0%		610	86.9%		412	58.7%	
Hispanic Black	2	13.30%		0	0.0%		13	76.5%		11	64.7%	
Other	12	9.60%		9	7.1%		112	88.9%		84	66.7%	
Language												
English	183	12.0%	0.93	80	5.3%	0.47	1,319	86.8%	0.35	925	60.9%	0.14
Spanish	29	12.4%		9	3.8%		212	90.2%		127	54.0%	
Other	5	12.8%		3	7.7%		35	89.7%		24	61.5%	

All patients included in the cohort received postoperative opioids, 763 (42.5%) received benzodiazepines, and 1,525 (85%) received dexmedetomidine. There were no differences in total doses of opioids, benzodiazepines, or dexmedetomidine according to patient race and ethnicity or patient preferred language (Table 3). These results are consistent with other studies of the interaction between race and ethnicity and doses of sedation during mechanical ventilation (Nathaly 2021), but no other study has evaluated the intersection of protocolized sedation and language or focused specifically on postoperative cardiac patients. There were also no differences in duration of mechanical ventilation, or rates of unplanned extubations, cardiac arrest or mortality according to patient race and ethnicity or language (Table 4).

**Table 3:** Doses of opioids, benzodiazepines, and dexmedetomidine in the first 72 h post-operatively according to patient race and ethnicity.

	Non-hispanic white		Non-hispanic black		Hispanic white		Hispanic black		Other			English		Spanish		Other		
	*n* = 706		*n* = 232		*n* = 702		*n* = 17		*n* = 126			*n* = 1520		*n* = 235		*n* = 39		
	Median	IQR	Median	IQR	Median	IQR	Median	IQR	Median	IQR	*p* value	Median	IQR	Median	IQR	Median	IQR	*p* value
Opioid (MME/kg)	1.9	0.9–5.4	2.1	0.8–6.1	2.1	1.0–6.5	1.4	0.8–3.2	2.4	0.8–7.0	0.57	2.1	0.9–5.8	1.9	0.9–7.0	2.5	0.8–8.3	0.69
Benzodiazepine (midaz mg/kg)	0	0–0.2	0	0–0.1	0	0–0.2	0	0–0	0	0–0.2	1	0	0–0.2	0	0–0.1	0	0–0.2	1
Dexmedetomidine (mcg/kg)	6.5	1.6–16.4	7.3	1.5–19.4	6.4	1.2–17.4	5.9	0–14.8	6	1.5–18.4	0.88	6.5	1.4–17.4	6.2	1.5–18.7	7.3	0.2–15.8	0.87

**Table 4:** Outcomes according to patient race, ethnicity and language.

	Non-hispanic white		Non-hispanic black		Hispanic white		Hispanic black		Other			English		Spanish		Other		
	*n* = 425		*n* = 138		*n* = 412		*n* = 11		*n* = 84			*n* = 1520		*n* = 235		*n* = 39		
	Median	IQR	Median	IQR	Median	IQR	Median	IQR	Median	IQR	*p* value	Median	IQR	Median	IQR	Median	IQR	*p* value
Duration of Mechanical Ventilation (hours)	22.5	10.9–51.5	23.4	17.4–75.5	21.8	10.9-67.2	10	5.8–17.5	22.4	10.2–68.9	0.64	22.0	10.9–65.6	23.1	13.6–73.1	21.3	15.3–45.1	0.92
	*n*	%	*n*	%	*n*	%	*n*	%	*n*	%		*n*	%	*n*	%	*n*	%	
Unplanned Extubation	7	1.0%	1	0.4%	6	0.9%	0	0%	2	1.6%	0.74	14	0.9%	1	0.4%	1	2.6%	0.26
	*n* = 706		*n* = 232		*n* = 702		*n* = 17		*n* = 126			*n* = 1520		*n* = 235		*n* = 39		
Cardiac Arrest	8	1.1%	1	0.4%	9	1.3%	0	0%	3	2.4%	0.51	15	1.0%	5	2.1%	1	2.6%	0.16
Mortality	17	2.4%	3	1.3%	20	2.8%	0	0%	4	3.2%	0.68	37	2.4%	6	2.6%	1	2.6%	0.87

Race and ethnicity, language, and insurance were not associated with total doses of opioids, benzodiazepines, or dexmedetomidine in unadjusted or multivariable quantile regression. There were several significant associations in unadjusted regression that would be clinically expected including higher doses of opioids and benzodiazepines for patients who were mechanically ventilated (coefficient 2.21, *p* < 0.05), had longer hospital length of stay (coefficient 0.03, *p* < 0.05), and underwent DSC (coefficient 7.19, *p* < 0.05). Dexmedetomidine demonstrated similar findings with lower doses for older age (coefficient −0.26, *p* < 0.05) and higher doses for patients who were mechanically ventilated (coefficient 5.95, *p* < 0.05), had longer hospital length of stay (coefficient 0.06, *p* < 0.05), and underwent DSC (coefficient 10.55, *p* < 0.05). Multivariable regression with age, STAT category, and post-operative cardiac arrest yielded similar results.

## Discussion

Our study found no association between race, ethnicity, or language, and the total dose of opioids, benzodiazepines, and dexmedetomidine received in pediatric patients in the first 72 h following cardiac surgery. This study contributes uniquely to existing literature by focusing specifically on postoperative pediatric cardiac patients and consideration of patient-preferred language in comparisons. The observed variances in medication amounts align with clinical expectations based on illness severity and the typical postoperative trajectory involving mechanical ventilation, DSC, and longer LOS.

While numerous studies have highlighted disparities in analgesia administration based on race, ethnicity, and language, our study did not replicate these findings. This discrepancy is likely attributed to stringent adherence to standard sedation and analgesic protocols based on validated tools, which may have minimized implicit biases. Implicit biases in assessment of pain or sedation needs may still be influenced by the presence of family at the bedside, family health literacy, patient and provider racial or language concordance, and pre-operative illness severity despite the use of protocolized sedation and analgesia.

Despite the widespread adoption of sedation protocols in many adult and pediatric ICUs, research has failed to demonstrate a consistent effect of their impact on total medication dosages, duration of mechanical ventilation, ICU and hospital length of stay, or mortality rates as noted in a Cochrane review (23). Future investigations should extend beyond the acute postoperative period and focus on patients with prolonged hospitalizations in multicenter studies. Furthermore, it will be important to evaluate the efficacy of protocols in managing patients undergoing advanced treatment modalities such as renal replacement therapy and mechanical circulatory support. Centers currently not employing standardized management could benefit from comprehensive analyses comparing pre- and post-implementation outcomes.

Our study found no differences in duration of mechanical ventilation, rates of unplanned extubations, cardiac arrest, or mortality. These findings were unexpected and are in contrast to much of the published data regarding disparities in ICU outcomes according to race, ethnicity, and language. This may be due to the narrow scope of the study population, the variation in patient reported demographics from the study site population, adherence to strict postoperative care standards, and the study site being a large, high volume center for cardiac surgery. Further investigation in this realm is warranted to determine if there are factors that could be replicated in other settings to eliminate common disparities.

Limitations inherent in retrospective EMR data should be acknowledged, along with the absence of real-time decision-making details. Additionally, despite the large study sample size, the racial, ethnic, and language subgroups varied significantly in size and are not representative of the general population of the study site. This could result in selection bias or be reflective of the inadequacy of the current methods for reporting self-identified race and ethnicity and preferred language. While insurance is often used as a proxy for patient socioeconomic status, it lacks nuance and specificity that other markers of socioeconomic status may provide. Notably, this study period coincided with the global COVID pandemic, during which visitation restrictions were in place, yet medication dosages remained consistent, underscoring the robustness of the sedation practices. Nevertheless, it is essential to recognize the limitations of historical data, particularly regarding self-reported or assigned language and ethnicity, which may not fully capture the diversity within these categories or truly reflect patients' racial identities. Additionally, acknowledging race as a social construct underscores the need to consider the multifaceted social and environmental factors influencing health outcomes. Our study may also lack generalizability given it was a single center study with robust sedation and analgesia protocols, a racially, ethnically, and linguistically diverse patient and provider cohort, in a diverse U.S. city.

Overall, our study suggests that protocolized sedation should be further studied as a potential strategy to mitigate racial and language biases among postoperative pediatric cardiac patients admitted to the ICU. Further study is needed to better understand the interplay between sedation protocols and race and ethnicity biases longitudinally and at a multicenter level.

## Data Availability

The raw data supporting the conclusions of this article will be made available by the authors, without undue reservation.
